# Vascular vertigo and dizziness: managing and treating outpatients^☆^

**DOI:** 10.1016/j.bjorl.2024.101453

**Published:** 2024-06-06

**Authors:** Arlindo Cardoso Lima Neto, Roseli Saraiva Moreira Bittar

**Affiliations:** Setor de Otoneurologia do Hospital das Clínicas da Faculdade de Medicina da Universidade de São Paulo – FMUSP, Brazil

**Keywords:** Vascular dizziness, Vertigo dizziness, Stroke, Transient ischemic attacks, Vertebrobasilar insufficiency

## Abstract

**Objective:**

Due the lack of data on the treatment of Vascular Vertigo and Dizziness, this study aimed to report how we managed and treated those outpatients according to the recently introduced American Heart Association and Stroke Association guidelines.

**Methods:**

We conducted a longitudinal case series from May 2022 to February 2023. We included patients who met the Bárány Society’s Vascular Vertigo and Dizziness classification and were eligible for therapy in accordance with the American Heart Association and Stroke Association guidelines, featuring aspects of the stroke group and transient attack group.

**Results:**

Overall, 41 patients (51.2% female; median age 72 years) were enrolled; 10 (24.3%) had ischemic strokes, 30 (73.1%) had transient ischemic attack, and 1 (2.4%) had a probable isolated labyrinthine infarction. The patients received dual antiplatelet (48.7%), single antiplatelet therapy (48.7%), and anticoagulant therapy (2.4%). No new crises occurred in 95.2% of the patients, and the transient ischemic attack group showed a significant decrease in discomfort from imbalance on the visual analog scale.

**Conclusions:**

Antiplatelets and anticoagulants are safe and effective in treating Vascular Vertigo and Dizziness as they prevent new ischemic events and increase the flow of the posterior circulation, reducing vertigo/dizziness attacks and imbalance complaints.

**Level of evidence:**

4.

## Highlights

• Imbalance can be the main ischemic symptom on posterior circulation.
• Vascular vertigo or dizziness are associated to stroke and transient attacks.
• Antiplatelets are an important tool for treating vascular vertigo/dizziness.
• Dual antiplatelet therapy can be used in selected patients.
• Besides prevention, antiplatelets reduce the number of vertigo attacks.

## Introduction

The number of strokes has been steadily increasing due to the aging of population and modern lifestyle.^1^ A fifth of all Transient Ischaemic Attacks (TIAs) and ischaemic strokes are in the territory of the vertebrobasilar (also known as posterior) circulation, where dizziness and vertigo are listed in the top of other brainstem and cerebellum’s symptoms. Despite their importance, ischaemic events in this location have received much less attention than those in the carotid artery territory, and many authors agree that more prospective studies in this field are required.^2^, ^3^

Dizziness or vertigo can be the only symptom of Posterior Circulation (PC) ischemia,^4^, ^5^ although it is usually accompanied by other neurological symptoms.^6^ Recently, diagnostic criteria for Vascular Vertigo and Dizziness (VVD) were presented by the Bárány Society’s Committee for the Classification of Vestibular Disorders, making recognition of this disease more tangible and practical, and even defining stroke, TIA and isolated labyrinth infarction as part of this condition.^7^ Other recent milestone was the Guidelines for the Prevention of Stroke in Patients with Stroke and TIA, launched by American Heart Association and American Stroke Association (AHA/ASA), supported by an extensive evidence review. These guidelines highlight specific recommendations for prevention strategies for each ischemic stroke or TIA subtype, including those involving PC and narrowing of the vertebral artery.^8^ However, no studies have been conducted on the application of these new concepts and therapeutic recommendations regarding VVD patients, nor prospective cohorts describing their evolution and prognosis.

Although Emergency Department (ED) is the ideal care setting for managing VVD crises, many patients seek office appointments that rarely occur on the same day. This results in other challenges because there is a paucity of data reported on VVD outpatients. Considering the clinical aspects and temporal gap between the ischemic event and appointment, many approaches should be reviewed and adapted to manage and treat these patients out of ED.

This study is based on a historical cohort that our group has been building over the years, to furnish data on all the areas that VVD’s literature lacks. Our goal is to report our outpatient experience based on the new AHA/ASA guidelines. We intend to describe the six-month follow-up in relation to the number of crises, occurrence of stroke and therapeutic outcome.

## Methods

### Patient selection

A prospective cohort was obtained from our outpatient neurotology clinic between May 2022 and February 2023.

Patients were included based on two criteria:

• The diagnostic criteria for VVD by the Committee for the Classification of Vestibular Disorders of the Bárány Society were fulfilled. At least one of the crises had to qualify as “confirmed or probable acute prolonged VVD”, “confirmed or probable transient VVD”, or “vertebral artery compression syndrome”. Patients experiencing an acute episode during the appointment were referred to the ED; hence, “confirmed or probable acute vascular vertigo/dizziness in evolution” was not considered.^7^
• Patients needed to be eligible for antiplatelet or anticoagulant therapy in agreement with the Guidelines for the Prevention of Stroke in Patients with Stroke and TIA ‒ AHA/ASA 2021; that is, those who began single antiplatelet, single anticoagulant, or Dual Antiplatelet Therapy (DAPS). Volunteers who had already taken one of these drugs and should maintain the same pattern because of VVD were not eligible.

Patients who fulfill diagnostic criteria for other neurotologic disease (benign paroxysmal positional vertigo, Menière’s disease, vestibular migraine, vestibular paroxysmia, vestibular neuritis, persistent postural-perceptual dizziness, postural hypotension, presbyvestibulopathy, motion sickness and cervical dizziness), neurodegenerative disorders and cognitive deficits were excluded, as previously stated in the Barany’s document.^7^

**Figure 1:**
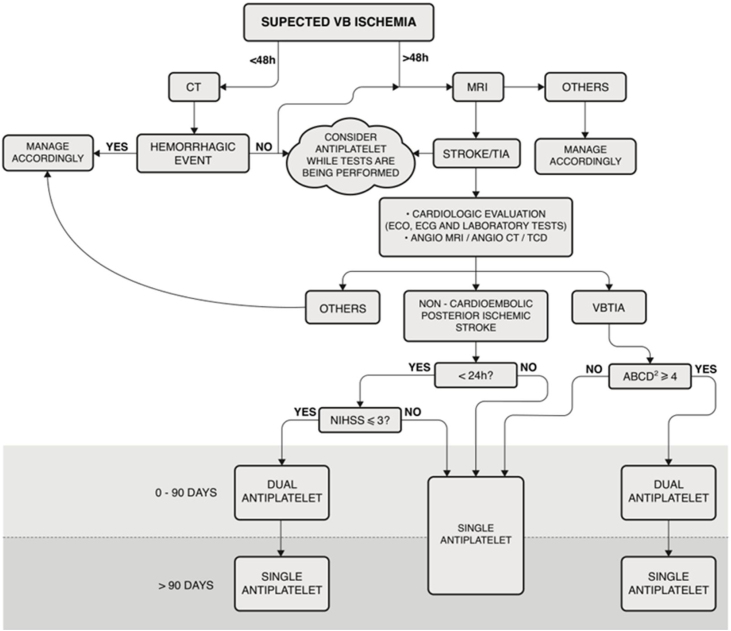
Algorithmic management of Vascular Vertigo/Dizziness in outpatients. Note 1: For all patients, vascular risks included lifestyle factors, hypertension, hyperlipidemia, glucose, obesity, and obstructive sleep apnea. Note 2: This algorithm is not applicable to patients receiving acute thrombolysis. MRI, Cranial diffusion-weighted Magnetic Resonance; CT, Cranial Computed Tomography; ECO, Echocardiography; ECG, Electrocardiogram. Laboratory tests included complete blood count, troponin, prothrombin time, partial thromboplastin time, glucose, hemoglobin A 1c, creatinine, and fasting and non-fasting lipid profiles. Angio-MRI, cranial and cervical Magnetic Resonance Angiography; Angio-CT, Cranial and Cervical Computed Tomography; TCD, Transcranial Doppler Ultrasonography; VBTIA, Vertebrobasilar; TIA, Dual Antiplatelet means aspirin + clopidogrel; NIHSS, National Institutes of Health Stroke Scale.

To confirm the diagnosis of VVD and exclude any possible others, all the participants underwent complete otorhinolaryngological and neurological evaluations. The tests included anamnesis, bedside tests (HINTS), positional vertigo/nystagmus, Video Oculographic examination (VOG), tonal and vocal audiometry and tympanometry. In some cases, the Vestibular-Evoked Myogenic Potential (VEMPs) and Video Head Impulse Test (VHIT) were also performed. Additionally, cardiovascular ABCD2 risk score data were collected. On suspicion of cardiologic or autonomic disease, ambulatory blood pressure monitoring, Holter electrocardiography, Doppler echocardiography and tilt tests were performed. All participants underwent Diffusion-Weighted Magnetic Resonance Imaging (MRI-DWI) to diagnose stroke and at least one Posterior Circulation Blood Flow Test (PCBFT) to assess Vertebrobasilar (VB) flow, which included Magnetic Resonance Angiography (MRA), Computed Tomographic Angiography (CTA), and Transcranial Doppler ultrasound (TCD). Fig. 1 shows the algorithm used to manage the outpatients in this sample.

### Therapeutic intervention

The first-line treatment for outpatients with VVD was aspirin (100 mg/day) or clopidogrel (75 mg/day), depending on allergy, bleeding, preference, or any other reported side effects. The DAPS was used for a maximum of 90 days, as shown in Fig. 1. In cases where patients were already taking an antiplatelet drug (aspirin or clopidogrel) and had a clinical indication for DAPS, aspirin plus clopidogrel was prescribed. When the most appropriate treatment was an anticoagulant, it was used as a monotherapy. Vascular risk factors such as lifestyle, hypertension, blood lipid and glucose levels, obesity, and obstructive sleep apnea were also monitored.^8^, ^9^, ^10^, ^11^

After treatment outset (day 0, D0), patients were followed up on the 30^th^ day (D30), 60^th^ day (D60), 90^th^ day (D90), and 180^th^ day (D180), when the Visual Analog Scale (VAS) of discomfort from imbalance and side effects were assessed. If a patient experienced a new attack during this process, the algorithm was restarted.

If a patient reported an imbalance affecting their daily activities despite using the recommended antiplatelet/anticoagulant regimen or reported an insufficient improvement, they underwent Vestibular Rehabilitation (VR). In addition, if PCBFT showed narrowing of >50% (PCBFT+), the patient was referred to a vascular surgeon and interventional radiologist.

### Variables

The previous use of antiplatelet or anticoagulant medicine for another illness; ABCD2 score range; final diagnosis (stroke, TIA, or isolated labyrinthine infarction); PCBFT+; VAS range of discomfort from imbalance throughout the follow-up; insufficient improvement and side effects within antiplatelet/anticoagulant therapy; VR; VAS after VR; vascular surgeon and interventional radiologist management and VAS subsequently.

The ABCD2 score is a clinical prediction score ranging from 0 to 7, which assigns points based on the following five clinical factors: age 60 years or older = 1; blood pressure ≥140/90 = 1; clinical features (unilateral weakness = 2, speech disturbance without weakness = 1, any other symptom = 0); duration of symptoms (<10 min = 0, 10–59 min = 1, ≥60 min = 2); and diabetes = 1.^12^ A modified ABCD2 was used once the patients were out of a crisis. Therefore, all hypertensive patients were scored as “1”, even with normal blood pressure levels at their appointment.

A patient was considered to have insufficient improvement if the VAS score of discomfort caused by the imbalance did not decrease by at least 50% by D90 or D180, despite using an antiplatelet/anticoagulant plan.

### Ethical considerations

All participants voluntarily agreed to participate in the study and signed an informed consent form. The relevant ethics committee approved this study, and our methodology complied with the principles of the Declaration of Helsinki.

### Statistical analysis

Comparisons were made using Fisher’s test for categorical variables and the Mann-Whitney test for continuous variables. Statistical significance was set at *p* < 0.05, using R version 4.2.0 Copyright (C) 2022 (The R Foundation for Statistical Computing).

## Results

Overall, 507 patients were assessed between May 2022 and February 2023, and 41 were enrolled (51.2% female) with a mean/median (SD) age of 70.3/72 (±10.1), ranging from 40 to 84 years. All datasets are summarized in Table 1, Table 2.

**Table 1:** Patient characteristics, categorized by identification number, sex, age, symptoms, and imaging findings.

Id	Age	Sex	Other hypo flow symptoms	Other previous illnesses	Previous Antiplatelet/ Anticoagulation	ABCD2	Imaging findings	PCBFT+
1	80	M	Transient visual loss, syncope	AH, dyslip, arrhythmia	N	2	LV4 narrowing, non-ischemic MRI	Y
2	80	F	Transient visual loss	AH	N	2		N
3	78	M	New-onset tinnitus	AH, aortic valvuloplasty	Y	4	L cerebellar hemispheric stroke	N
4	72	M	Syncope	AH, diabetes	N	3		N
5	70	F	Transient visual loss, cervical paresthesia	AH, hypothyroidism, rheumatoid arthritis	N	2	LV1 narrowing, non-ischemic MRI	Y
6	60	F	Syncope, dysphagia	Crohn’s disease	N	2	RV Dissection, non-ischemic MRI	Y
7	55	F	Arm paresthesia	Diabetes	N	2	Bilateral multiple ischemia	N
8	67	F	Cranio-cervical paresthesia and temporary unilateral hearing loss	AH, diabetes, dyslip, hypothyroidism	N	4	RV1-4 narrowing, non-ischemic MRI	Y
20	55	M	Recurring BPPV at the left side, dysphagia		N	0	LV1-4 narrowing, non-ischemic MRI	Y
22	75	F	Syncope		N	2	RV1-4 narrowing, non-ischemic MRI	Y
23	71	F		Arrhythmia, dyslip	N	2		N
24	67	F	Balance-related fall, syncope	Diabetes, hypothyroidism, rheumatoid arthritis	N	3		N
25	83	M	Syncope, cranial pain	AH, coronary insufficiency	Y	4	LV1 narrowing, non-ischemic MRI	Y
26	51	M	Balance-related fall, dysphagia, dysarthria, transient visual loss	Stroke, AH, diabetes, dyslip	Y	2	Basilar narrowing, R cerebellar hemispheric stroke	Y
27	77	M	Transient visual loss, Syncope	Stroke, coronary insufficiency, AH, diabetes, dyslip	Y	4	RV1-4 narrowing, R cerebellar hemispheric stroke	Y
28	67	F	Transient visual loss	AH, hypothyroidism, diabetes, dyslip	Y	3		N
29	76	F	Truncal ataxia, new onset of craniocervical pain, dysphagia	Stroke, AH, diabetes, dyslip	Y	6		N
30	63	M	New onset of cranial pain	Hypothyroidism	N	3		N
31	75	M	Diplopia, amnesia	Diabetes	Y	4		N
32	64	M	Diplopia	Diabetes, Hypothyroidism, dyslip	Y	3		N
33	69	M	New-onset of left-cranial-pain adn -tinnitus	AH	N	4	L cerebellar hemispherc stroke	N
34	72	M	Transient visual loss	AH, diabetes, intracranial hemorrhage	N	4		N
35	83	F	New onset of cranial pain, syncope	AH, diabetes, hypothyroidism	N	6		N
36	71	M	Transient visual loss, syncope	AH, diabetes	N	4	RV1-4 narrowing, non-ischemic MRI	Y
37	60	M	Balance-related fall, transient visual loss	AH, dyslip, hypothyroidism, rheumatoid arthritis	N	2		N
38	73	F	Hemiparesis	Arrhythmia, dyslp, diabetes, osteoporosis	N	3		N
39	80	F	Balance-related fall, hemiparesis	HA, hypothyroidism, arrhytmia (AF)	Y	4		N
40	58	M	Facial paresthesia, dysphagia, transient visual loss	AH, diabetes, dyslip	N	3	L multiple ischemia	N
41	60	F	Balance-related fall	Asthma, dyslip, osteoporosis	N	1		N
42	84	M	Sensory deficits	AH, dyslip, arrhythmia	N	3	LV4 narrowing, non-ischemic MRI	Y
43	72	F	Transient visual loss, syncope		N	2		N
44	72	F	New-onset of cranial pain, balance-relate fall	AH, diabetes, dyslip, hypothyroidism	N	3	RV1-4 narrowing, bilateral multiple ischemia	Y
45	81	F	Truncal ataxia, syncope	AH, diabetes, dyslip, hypothyroidism, osteoporosis	Y	3	RV2-3 narrowing, non-ischemic MRI	Y
46	78	F	Transient visual loss, balance-related fall, facial paresthesia	AH, dyslip, arrhythmia, hypothyroidism	N	2		N
54	40	F	New onset of cranial pain, sensory deficits	AH	N	5	RV Dissection, R pontine stroke	Y
49	79	M	Transient visual loss, balance-related fall, syncope	AH, diabetes, coronary insufficiency, thalassemia minor, silicosis	N	3		N
50	80	M	Syncope	AH, diabetes, hyperuricemia	N	3	R pontine stroke	N
53	84	F	Diplopia, dysarthria	AH, diabetes, arrhythmia, hypothyroidism	Y	5	L cerebellar hemispheric stroke	N
51	72	F	Balance-related fall	AH, dyslip	N	3		N
52	72	M	Syncope		N	1		N
56	58	M	Truncal ataxia		N	2		N

Id, Identification; PCBFT+, Narrowing of >50% of an artery on the VB system; AH, Arterial Hypertension; Dyslip, Dyslipidemia; AF, Atrial Fibrillation; Y/N, Yes/No; R/L, Right/Left; V1‒V4, Vertebral artery segment.

**Table 2:** Patient characteristics featuring diagnosis, treatment, and follow-up data.

Id	Diagnosis	Treatment	VAS D0	VAS D30	VAS D60	VAS D90	VAS D180	II	VR	VAS-VR	S/I	VAS-S/I
1	TIA	Clopidogrel	5	0	0	0	0	N	N			
2	TIA	Clopidogrel	8	0	0	0	0	N	N			
3	Stroke	DAPS	5	1.2	1.2	1.2	1.2	Y	Y	0.3		
4	TIA	Clopidogrel	4	0	0	0	0	N	N			
5	TIA	Clopidogrel	5	0.6	0.6	0.6	0.6	N	Y	0		
6	TIA	Clopidogrel	10	5	5	5	0	N	N			
7	Stroke	DAPS	7	0.7	0.7	0.7	5	N	Y	ig		
8	LI	Clopidogrel	3	0.3	0.3	0.3	0.3	N	N			
20	TIA	Aspirin	3	0	0	0	0	N	N			
22	TIA	Clopidogrel	7	0	0	0	0	N	N			
23	TIA	Aspirin	10	0	0	0	0	N	N			
24	TIA	DAPS	10	5	5	Ig	ig	N	N			
25	TIA	DAPS	9	9	9	9	9	Y	Y	ig	LV1 stent	9
26	Stroke	DAPS	10	10	10	10	10	Y	N		Expect	
27	Stroke	DAPS	5	5	5	5	5	Y	N		Expect	
28	TIA	DAPS	8	6	4	4	4	N	N			
29	TIA	DAPS	10	0	0	0	0	N	N			
30	TIA	DAPS	6	2.4	1.2	0	0	N	N			
31	TIA	DAPS	5	1.5	0	0	0	N	N			
32	TIA	DAPS	10	0	0	0	0	N	N			
33	Stroke	DAPS	10	8	5	2	2	N	N			
34	TIA	DAPS	8	0	0	0	0	N	N			
35	TIA	DAPS	10	0	0	0	0	N	N			
36	TIA	Clopidogrel	5	0	0	0	0	N	N			
37	TIA	Clopidogrel	8	2.4	2.4	1.6	ig	ig	N			
38	TIA	Clopidogrel	5	0	0	0	ig	ig	N			
39	TIA	Rivaroxaban^a^	6	0	0	0	0	N	N			
40	Stroke	DAPS	5	2.5	2.5	8	8	Y	Y	8		
41	TIA	Clopidogrel	10	7	2	2	ig	ig	N			
42	TIA	Clopidogrel	6	3.6	3.6	3.6	3.6	Y	Y	ig		
43	TIA	Clopidogrel	10	1	ig	Ig	ig	ig	N			
44	Stroke	DAPS	5	3	3	3	ig	Y	N		Expect	
45	TIA	DAPS	10	0	0	0	0	N	N			
46	TIA	Aspirin	6	0	0	0	0	N	N			
54	Stroke	Aspirin	10	0	0	0	0	N	N			
49	TIA	DAPS	10	5	ig	Ig	ig	N	N			
50	Stroke	DAPS	0	0	0	0	ig	ig	N			
53	Stroke	DAPS	10	2.5	1	0	ig	N	N			
51	TIA	Aspirin	3	2	2	Ig	ig	N	N			
52	TIA	Aspirin	7	5	ig	Ig	ig	ig	N			
56	TIA	Clopidogrel	5	3	ig	Ig	ig	ig	N			

VAS, Visual Analog Scale; II, Insufficient Improvement; VR, Vestibular Rehabilitation; S/I, Vascular Surgeon and Interventional radiologist assessment; D0, D30, D60, D90, and D180, days of follow-up; VAS-VR, VAS after VR; VAS-S/I, VAS after S/I; LI, Probable Isolated Labyrinthine Infarction; DAPS, Dual Antiplatelet Therapy; Y/N, Yes/No; ig, unknown data; LV1, first segment of the Left Vertebral artery; Expect, Expectant management.

a Rivaroxaban is the only anticoagulant used in this dataset.

Ten patients (24.3%) had MRI-DWI findings indicating ischemic stroke, 5 hemispheric cerebellar (posterior inferior cerebellar artery territory), 3 multiple ischemic findings (involving PC and middle cerebral artery), and 2 pontine strokes (basilar artery territory) (Table 1). One patient (2.4%) had a probable isolated labyrinthine infarction, and 30 (73.1%) had TIA.

The follow-up period was 180 days for 29 patients (70.7%), 90 days for 35 patients (85.3%), and 60 days for 37 patients (90.2%).

The ABCD2 score ranged from 0 to 6, with a mean ± SD of 3/3 ± 1.3 P.

Fourteen patients (34.1%) had significant (>50%) narrowing of VB system (PCBFT+).

Twenty patients (48.7%) were treated with DAPS, 20 (48.7%) with a single antiplatelet agent, and 1 (2.4%) with anticoagulant therapy (rivaroxaban) due to atrial fibrillation (patient 39). Additionally, 11 patients (26.8%) previously used antiplatelet drugs for other illnesses. Among the patients who required DAPS, 10 (50%) had previously received antiplatelet monotherapy.

Table 3 shows the frequency of vertigo/dizziness attacks after therapy initiation, which was also related to the diagnosis.

**Table 3:** Frequency of vertigo/dizziness attacks related to final diagnosis (after treatment).

Frequency of attacks	Diagnosis			Total
	Stroke	TIA	LI	
Single episode	5 3, 33, 54, 50, 53	5 31, 35, 46, 51, 56	1 8	11
1‒4 month	1 40	17 2, 4, 5, 20, 22, 23, 24, 29, 34, 38, 39, 41, 42, 43, 45, 49, 52	0	18
>4 month	4 7, 26, 27, 44	8 1,6,25,28,30,32,36,37	0	12
Total	10	30	1	41

Small numbers indicate patient identification. TIA, Transient Ischemic Attack; LI, Probable Iolated Labyrinthine Infarction.

**Figure 2:**
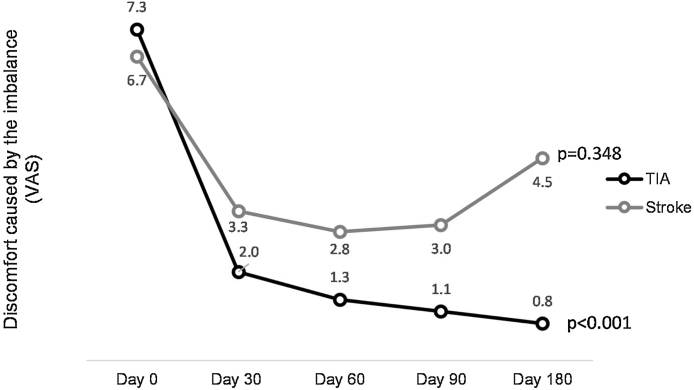
Visual Analogue Scale (VAS) for imbalance discomfort over follow-up days in the stroke and TIA groups.

Fig. 2 illustrates the variation in discomfort from imbalance throughout the follow-up within antiplatelet therapy, both in the stroke and TIA groups. There was a significant decrease in the VAS score in the TIA group between D0‒D30 and D0‒D180 (*p* < 0.0001), but no significant change between D30‒D180 (*p* = 0.1). However, there were no significant differences in the stroke group (*p* = 0.348).

Seven patients reported insufficient improvement with antiplatelet therapy. Table 4 shows that it was more prevalent in the stroke group than in the TIA group (*p* = 0.018), but there was no correlation with PCBFT+ and previous antiplatelet therapy. There was no significant difference comparing ABCD2 score among patients who reported insufficient improvement (mean = 3.3, SD = 0.8) and the other participants (mean = 3.2, SD = 1.4) (*p* = 0.691).

**Table 4:** Insufficient improvement within antiplatelet therapy and posterior circulation blood flow tests with narrowing >50% (PCBFT+) (*p* = 0.097), previous antiplatelet therapy for another illness (*p* = 0.186) and final diagnosis (*p* = 0.018).

	Insufficient improvement of balance	
**PCBFT+**	**No**	**Yes**
No	18 (66.7%)	2 (28.6%)
Yes	9 (33.3%)	5 (71.4%)
**Previous Antiplatelet therapy**	**No**	**Yes**
No	19 (73.1%)	3 (42.9%)
Yes	7 (26.9%)	4 (57.1%)
**Diagnosis**	**No**	**Yes**
TIA	20 (87.0%)	2 (33.3%)
Stroke	3 (13.0%)	4 (66.7%)

**Figure 3:**
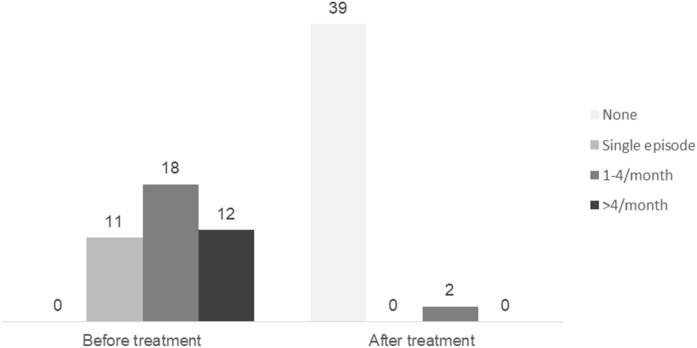
Frequency of vertigo/dizziness attacks after and before antiplatelet therapy (*p* < 0.001).

Fig. 3 and Table 5 show the changes in the frequency of vertigo/dizziness attacks before and during the treatment. Only two patients reported the recurrence of ischemic crises (patient numbers 25 and 26), and both had PCBFT+. They underwent a new MRI, which showed no differences from the previous one.

**Table 5:** Frequency of vertigo/dizziness attacks after and before the treatment (*p* < 0.001).

Frequency of attacks	Time	
	Before treatment	After treatment
None	0 (0.0%)	39 (95.1%)
Single episode	11 (26.8%)	0 (0.0%)
1‒4 month	18 (43.9%)	2 (4.9%)
>4 month	12 (29.3%)	0 (0.0%)

No patients reported any side effects with the proposed therapy during follow-up.

Despite some improvement, six patients (14.6%) maintained some level of discomfort from the imbalance and underwent Vestibular Rehabilitation (VR). Two of them (patients 3 and 5) showed a considerable decrease in VAS, whereas VAS in patient 40 remained unaltered, and three patients (7, 25, and 42) did not return until the conclusion of this dataset.

Four PCBFT+ patients (9.7%) who reported insufficient improvement were referred for vascular surgeon and interventional radiologist. Patient 25 had a stent placed on the left vertebral artery (V1 segment) without any improvement in his imbalance, despite the absence of cranial pain. The others three patients received expectant management from experts.

## Discussion

Although our neurotology clinic has been managing patients with VVD for over four decades, the recent Bárány Society’s Committee for the Classification and the Guidelines from AHA/ASA have introduced new models for the nomination, classification, management, and treatment of these patients, which motivated us to catalog and publish our results.

We presented a sample with a balanced sex distribution and age, similar to VVD studies performed in ED settings^13^, ^14^ and studies involving both PC and anterior circulation strokes.^15^ The sample had a low risk of ischemic stroke according to the ABCD2 score, only approximately 1/3 PCBFT+, and the most prevalent final diagnosis was Vertebrobasilar TIA (VBTIA), which may be biased due to the study being conducted in an otorhinolaryngological clinic, where the number of strokes is low.

The ABCD2 score is a simple, validated, and widely applied clinical prediction tool for assessing the risk of stroke after TIA,^12^, ^16^ and many studies have applied it to VVD.^17^ Our results did not demonstrate a correlation between mild or high ABCD2 scores and a VVD diagnosis. When designing our study, assuming a modified ABCD2 using “arterial hypertension = 1” instead of “blood pressure”, we presumed a possibly overrated score; however, it was not reflected in our results. Thus, it appears that ABCD2 has some bias towards anterior circulation events, mainly the 2-score for “unilateral weakness”. Several other studies have discussed the limitations of the ABCD2 and even proposed scoring systems to evaluate VVD;^13^, ^14^, ^18^, ^19^ nevertheless, most lack validation. Hence, future studies are needed to elucidate this weakness.

Our hospital is a tertiary referral center where imaging tests and cardiologist evaluations often take a few days.^20^ Fig. 1 shows that in our algorithmic management, we consider starting antiplatelet therapy even while tests are being conducted because of the higher risk of recurrence or a new, larger ischemic event, as shown by many studies.^18^, ^21^, ^22^, ^23^, ^24^

Consistent with these data, Table 1, Table 3 show that most participants reported multiple episodes of vertigo/dizziness associated with other hypo flow symptoms such as dysphagia, syncope, transient visual loss, and new headache onset. We highlight those five patients with stroke (50%) reported several crises, and it is unclear whether it was a TIA or an established stroke.^7^

Among the 30 patients with TIA, 25 (83.3%) referred more than one attack, indicating the same pattern of recurrence. Despite the lack of consensus regarding VBTIA, several studies have cited vertigo as the most frequent ischemic symptom.^25^, ^26^, ^27^ In patients with definite VB stroke, preceding transient isolated brainstem symptoms are common (as vertigo or dizziness), but most symptoms do not meet criteria for TIA.^3^, ^6^, ^7^ Our sample shows that only 1/30 TIA patient (Patient 23) had no other hypo flow symptom. She is a 71-years-old lady with arrhythmia, diabetes and dyslipidemia. Her crisis completely ceased after using aspirin. In a prospective, population-based incidence study in Oxfordshire, UK (Oxford Vascular Study), of all 59 patients with transient brainstem symptoms preceding vertebrobasilar stroke, only five fulfilled the official criteria for VBTIA. The other 54 cases comprised isolated vertigo (n = 23), binocular visual disturbance (n = 9), vertigo with other non-focal symptoms (n = 10), isolated slurred speech, hemisensory tingling or diplopia (n = 8), and non-focal events (n = 4).^3^ To identify these symptoms quickly, sometimes based on a diagnosis of exclusion and risk factors, is a challenge for clinicians to achieve rapid access to secondary prevention after TIA.^2^, ^3^, ^7^

Our most important finding was that the AHA/ASA proposal, which was intended for secondary prophylaxis of recurrence ischemic events, led to effective VVD therapy for 39/41 (95.2%) patients whose vertigo/dizziness attacks ceased because of increased blood flow, (Figure 2, Figure 3, Table 5). Only two participants (4.8%), patient 25 (90% narrowing of the left vertebral artery on segment V1 and multiple VBTIAs) and patient 26 (narrowing of the basilar artery and right hemispherical cerebellar stroke, in addition to many VBTIA), maintained regular ischemic episodes despite using DAPS, probably because of the severity of their PC obstruction. None of the patients developed a new stroke during the 6-month follow-up. Population-based studies have shown that approximately one-third of the first TIAs occur 2-weeks prior to the presenting stroke, and given the short time window between TIA and stroke, all patients with TIA should be treated urgently.^23^, ^28^, ^29^ Therefore, we considered this therapeutic management successful.

In addition to the considerable decrease in the number of crises, we observed a significant decrease in the discomfort level attributed to the imbalance measured by the VAS on each day of follow-up. Fig. 2 shows that despite the absence of new crises, there were no significant changes in VAS scores among stroke patients, which may be due to sequelae in the balance centers of the cerebellum and brainstem. In contrast, the TIA group showed significant improvement in the early weeks. Upon examining the graph for TIA patients, we observed a downward curve over time; however, we only found statistically significant differences between D0‒D30 and D0‒D180 (*p* < 0.0001), but not between D30‒D180. Nevertheless, our clinical perception tends to pay attention to these data as some patient complaints, such as positional instability or transient lightheadedness, were not addressed in the draft methodology but seemed to improve progressively, possibly due to the increased blood flow to the cerebellum and brainstem. This may explain why the VAS score appeared to be lower during follow-up. Further studies are required to address this issue.

Long-term use of DAPS is not recommended, and short-term dual antiplatelet therapy is recommended only in very specific patients, including those with early arriving minor stroke and high-risk transient ischemic attack or severe symptomatic intracranial stenosis.^8^ Although DAPS was an important tool in this study, it was received by 20/41 patients, and we highlighted that 10/20 patients with DAPS had previously used a single antiplatelet agent for another disease. Furthermore, many other drugs have been recommended to manage TIA and ischemic stroke, including ticagrelor, cilostazol, and dipyridamole.^30^, ^31^, ^32^ However, considering the higher recommendation class and level of evidence of aspirin/clopidogrel/DAPS,^8^ we selected them as the first-line therapy in our study.

Anticoagulation therapy is recommended for patients with stroke or TIA, regardless of cardioembolic conditions.^8^, ^33^, ^34^ Several patients in our dataset had previously taken anticoagulants and were excluded, except for patient 39, who was recently diagnosed with atrial fibrillation. In consultation with the cardiologist, we prescribed rivaroxaban.^35^

Bleeding is the most significant antiplatelet-associated side effect. As the bleeding risk with aspirin monotherapy is dose-dependent, aspirin doses should be kept as low as possible. Clopidogrel has a bleeding risk similar to that of aspirin, although a reduced incidence of gastrointestinal bleeding events suggests lower gastrotoxicity.^36^ However, none of the patients in this study presented with any side effects.

Seven participants had insufficient improvement, and this outcome was strongly associated with the diagnosis of stroke and its consequences, although there was no correlation with ABCD2, previous antiplatelet use, or PCBFT+. Among these patients, six underwent VR, of which two achieved a considerably lower VAS, one was at the same level (patient 25), and three did not return before the conclusion of this dataset. This result was predicted when the rehabilitation program improved gait and dynamic balance by acting on the vestibular system as a facilitator of recovery.^37^

Among the patients who had insufficient improvement, four with PCBFT+ were referred to a vascular surgeon and an interventional radiologist. One of them (Patient 25) had a stent placed in the left vertebral artery; however, there was no improvement in dizziness despite the increase in cranial pain. Even though this dataset contains a low number of stents, this result is consistent with those of several studies. These studies also suggested that the need for stent placement in preventing ischemic stroke in the territory of the stenotic artery is unknown for patients with severe stenosis (70%‒99%) of a major intracranial artery and recurrent TIA or stroke after the institution of DAPS. In contrast, the other three, after their referral, could not be categorized and entered into any subsequent medical treatment protocol.^8^, ^38^, ^39^

Of note, one critical but ethically unavoidable limitation of the study was that our dataset has no placebo group. To our defense, several trials have already demonstrated the efficacy of antiplatelets and anticoagulants in ischemic syndromes.^8^ Thus, we conducted this cohort trying to reduce the lack of data on antiplatelets/anticoagulants in VB circulation.

## Conclusion

In this cohort, we managed VVD outpatients based on the Guidelines for the Prevention of Stroke in Patients with Stroke and TIA-AHA/ASA. However, we considered prompt and safe initiation of antiplatelet/anticoagulant therapy to increase the flow of PC and, consequently, reduce vertigo/dizziness attacks and imbalance discomforts. VR and vascular surgeon or interventional radiologist assessments could be tools for patients reporting insufficient improvement.

There were no new crises in more than 95% of the participants, and none presented with new ischemic strokes or side effects at the six-month follow-up. Some minor symptoms, such as transient lightheadedness and positional instability, also decreased progressively, especially in patients with TIA.

## Funding

This research did not receive any specific grant from funding agencies in the public, commercial, or not-for-profit sectors.

## Conflicts of interest

The authors declare no conflicts of interest.
